# Long-term care, care needs and wellbeing of individuals after cancer in childhood or adolescence (VersKiK): study protocol of a large scale multi-methods non-interventional study

**DOI:** 10.1186/s12913-022-08549-3

**Published:** 2022-09-20

**Authors:** E. Aleshchenko, E. Swart, C. Spix, M. Voigt, P. Trocchi, T. Langer, G. Calaminus, K. Baust, J. Glogner, P. Ihle, J. Küpper-Nybelen, C. Lüpkes, T. Kloppe, D. Horenkamp-Sonntag, I. Meier, U. Marschall, P. Dröge, M. Klein, A. Weiss, C. Apfelbacher

**Affiliations:** 1 Institute of Social Medicine and Health Systems Research, Faculty of Medicine, Otto Von Guericke Univiersity, Magdeburg, Germany; 2 Division of Childhood Cancer Epidemiology, Institute of Medical Biostatistics, Epidemiology and Informatics, University Medical Center of Johannes Gutenberg University Mainz, Mainz, Germany; 3 University Hospital of Schleswig-Holstein, Campus Lübeck, Lübeck, Germany; 4 Department of Pediatric Hematology and Oncology, University Hospital Bonn, Bonn, Germany; 5 PMV Research Group at the Department of Child and Adolescent Psychiatry, Psychotherapy and Psychosomatics, University of Cologne, Köln, Germany; 6 OFFIS–Institute for Information Technology, Oldenburg, Germany; 7 Techniker Krankenkasse (TK), Hamburg, Germany; 8 BARMER, Wuppertal, Germany; 9 AOK Research Institute (WIdO), Berlin, Germany; 10 DAK-Gesundheit, Hamburg, Germany; 11 Medical Sociology, Institute for Epidemiology and Preventive Medicine, University of Regensburg, Regensburg, Germany; 12 Bavarian Care and Nursing Authority, Amberg, Germany

**Keywords:** Cancer survivorship, Follow-up studies, Cancer, Late effect, Follow-up guidelines, Insurance claims processing, Transition to adult care, Informal caregivers

## Abstract

**Background:**

It has been shown previously that a relevant proportion of childhood cancer survivors suffers from late effects, which are often directly related to the cancer itself or its therapy, resulting in particular follow-up needs, additionally burdening healthcare systems. Being diagnosed with cancer at a vulnerable stage of development, this group of cancer survivors is at comparatively higher risk of relapse or subsequent cancer. Although national and international follow-up guidelines based on treatment modalities have been developed, their implementation seems to leave room for improvement. Additionally, they lack a sufficient consideration of the survivors’ psychosocial needs, affecting their adherence to them. The aim of the VersKiK study is to provide representative information on late effects in childhood and adolescence cancer survivors in Germany. The main research objectives are: (1) to describe the state of follow-up care among survivors after a cancer diagnosis in childhood or adolescence; (2) to quantify the occurrence of late effects among this group of survivors; (3) to examine the adherence to selected audiological and cardiological follow-up guidelines and to identify factors affecting it; (4) to explore actual follow-up needs of paediatric cancer survivors; (5) to review selected follow-up guidelines with the aim to improve and expand them.

**Methods:**

VersKiK is designed as a mixed-methods non-interventional study. We will use claims data from statutory health insurance companies in combination with individually linked population-based registry data from the German Childhood Cancer Registry (GCCR). This data base will permit us to quantify diagnoses and procedures in comparison to the general population as well as the adherence to existing follow-up guidelines. Additional information will be obtained through interviews with childhood and adolescence cancer survivors and their informal caregivers, as well as in focus groups with healthcare professionals.

**Discussion:**

The present study aims to research the actual needs of individuals after cancer diagnosis and treatment in childhood or adolescence – physical, psychological and organisational – in order to improve existing follow-up guidelines. These improvements might further positively affect not only actual care provided to paediatric cancer survivors, but also benefit healthcare systems in general while decreasing consequent medical visits in this group of patients.

**Trial registration:**

Registered at German Clinical Trial Register (ID: DRKS00025960 and DRKS00026092).

## Background

About 2100 children and young adults are diagnosed with cancer before their 18th birthday in Germany each year [1]. The survival of most childhood cancer has improved considerably in recent decades as diagnostics and treatments have further advanced. In Germany, currently more than 80% of all individuals diagnosed with a paediatric cancer survive 15 years or more after the initial diagnosis [2]. International research on late effects shows that around two-thirds of all childhood cancer survivors suffer from at least one late effect during their life, which is directly or indirectly associated with the cancer itself or its treatment [3–6].

Studying late-effects is crucial to better understand the risks to wellbeing of cancer survivors. One major challenge in the investigation of late effects is a fragmentation of data sources. A large proportion of data available originates either from interviews with cancer survivors [7–9] or from large, population-based cancer registries [10–16]. For Germany, only limited information on late effects of childhood cancer is available [17, 18].

Another issue affecting wellbeing of cancer survivors is a low adherence to follow-up. Recent evidence suggests that although specific guidelines exist [19–21], many survivors either are not taking advantage of them [22–24], or do not follow the guidelines to a sufficient extent [25, 26]. This might be caused by inadequacies in the consideration of the actual needs of childhood cancer survivors [27]. Previous studies have shown that the intention to attend follow-up is strongly affected by psychosocial factors, e.g. perceived barriers and beliefs towards health consequences [28] or fear due to past therapies [29]. Patients in transition from the childhood healthcare provider to the adult healthcare system have specific psychosocial needs [30–34], along with organisational difficulties caused by change from paediatric to adult care. In transition, patients’ intention to attend follow-up is also dependent on the involvement of informal caregivers [35–38]. For Germany, there is little systematic information regarding the adherence to follow-up guidelines or relevant predictors [39, 40].

Another factor contributing to reduced adherence might be the current focus of guidelines on care provision right after cancer treatment in order to prevent relapses [20, 21, 41–43]. This focus does not cover possible long-term late effects. Additionally, possible late-effects are not communicated in a standardized and age-adapted manner during protocol-based follow-ups.

Considering a necessity of life-long follow-up, reduced adherence results in an increased burden of morbidity, negatively affecting healthcare systems in general [44].

The overarching aim of this study is to comprehensively investigate the follow-up care and care needs of individuals who survived cancer in childhood or adolescence.

## Methods / design

VersKiK is a multi-method, non-interventional study organised in three modules. Table 1 shows objectives, study populations, data collection and methods for each of these modules.

**Table 1:** Study design

	Module 1	Module 2	Module 3
Study objectives	To describe the current state of the follow-up care among childhood and adolescence cancer survivors; To quantify the occurrence of late effects among this group of survivors	To explore actual follow-up needs of survivors of cancer in childhood or adolescence	To examine the adherence to selected audiological and cardiological follow-up guidelines and to identify factors affecting it; To review selected follow-up guidelines with the aim to improve and adapt them
Study population	GCCR patients (*N* = 46.200 individuals); matched comparison group of persons selected from the pool of insured persons of the participating statutory health insurance companies (expected *N* = 154,000 individuals)	Childhood and adolescence cancer survivors and their relatives – up to 30 patients; Healthcare professionals – up to 48 persons	GCCR patients with selected diagnoses and corresponding follow-up guidelines
Data collection	Data linkage of GCCR data and health insurance data based on cryptographed identity data via trust centres Comparison group: Matched random draw procedure from the pool of insured persons according to year of birth and gender of GCCR patients (relation: 1:5)	Episodic narrative interview; Instrumental case study; Focus group	Data linkage of GCCR data and health insurance data focused on diagnostic and therapeutic procedures for cardiological and audiological late effects in subgroups with available treatment data; Comparison of groups with different grade of guidelines adherence
Methods	Calculation of prevalence of late effects and frequencies of medical care claims in both the cohort of GCCR cancer cases and the comparison group; estimation of crude, matched and adjusted Prevalence Ratios (PR) using multiple log-linear regression models	Framework analysis	Calculation of prevalence of adherence to guidelines; estimation of crude and adjusted PR for late effects depending on degree of adherence using multiple log-linear regression models

The three modules are described in more detail in the following.

### Current state of the follow-up care and potential late adverse effects (module 1)

In order to describe a current state of the follow-up care and actual late adverse effects among survivors after a cancer diagnosis in childhood or adolescence, a retrospective cohort study will be conducted with cohorts of cancer survivors diagnosed between 1991 and 2022. The German Childhood Cancer Registry (GCCR) registers all cancer cases that are diagnosed in children under the age of 15 (since 2009: under 18 years) in Germany, covering > 95% of all childhood and adolescence cancer cases. All diagnoses are coded by ICD-O-3 and classified according to ICCC-3 [45]. Between 1991 (after German re-unification und the registry’s extension to the territory of the former German Democratic Republic) and 2022, the GCCR registered a total of 62,282 cases in children and adolescents born since 1 January 1976, of which approximately 46,200 were still alive and identifiable in Germany on 31 December 2016 and are therefore eligible for the study. Inclusion criteria are shown in Table 2.

**Table 2:** Inclusion criteria

	GCCR (registered new cases; identifiable patients)	Statutory health insurance companies (billing data for the matched cancer cases and a comparison group)
Inclusion criteria	- Date of birth from 1.1.1976; - Survivors until 31.12.2016; - Year of diagnosis from 1991 (registration in unified Germany); - ICCC-3 coded diagnoses; - Residence in Germany at the time of diagnosis and not living abroad on 31.12.2016; - Year of diagnosis 1991 to 2008: age at the time of diagnosis under 15 years; - From the year of diagnosis 2009: age at the time of diagnosis under 18 years	- Date of birth from 1.1.1976 - All insured persons who are alive on day of data extraction (30.09.2022) and those who died in the period between 1.1.2017 and the data extraction ( 30.09.2022) - Permanent residence in Germany for the period of data delivery

According to the current state of agreement, 13 statutory health insurance companies active in Germany, including the three largest ones, are going to participate in the study. Around 55 million individuals across all age groups are covered by the participating statutory health insurance companies in 2018, capturing around two thirds of the German population. Out of these, about 32% fall into the projects’ target age group. We therefore expect to include ca. 46,200*2/3 = 30,800 cases from the GCCR. In addition, ca. 154,000 (30,800*5) individuals will be included as comparison group. These two groups make up the study population of around 185,000 individuals. Inclusion criteria are presented in Table 2.

The participating statutory health insurance companies will provide information about a limited set of sociodemographic data, outpatient and inpatient medical care and claims of both the cancer case group and the comparison group, without knowing which group they belong to. The GCCR will provide baseline cancer information for the paediatric cancer survivor. Moreover, University Clinic Bonn (UKB) in collaboration with the dedicated clinical trials will provide treatment data for a selected subset of cancer survivors.

**Fig. 1:**
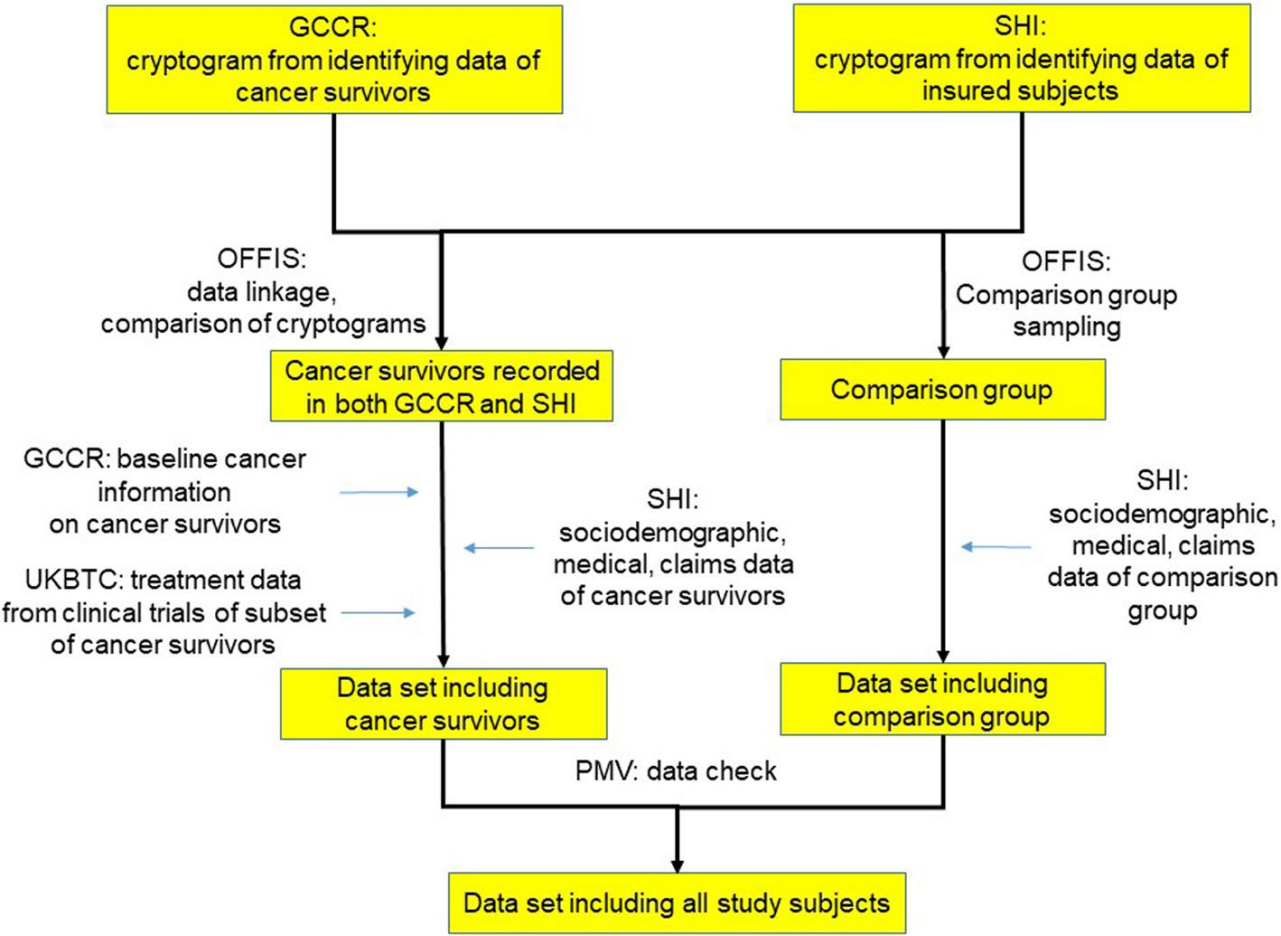
Step-by-step procedure for the preparation of the study data set (module 1). Legend: GCCR: German Childhood Cancer Registry, SHI: statutory health insurances, UKBTC: University Clinic Bonn in collaboration with clinical trials, PMV research group at the University of Cologne

The linkage steps, ID-data and medical data, are performed by two independent trust centres, OFFIS Oldenburg and the PMV research group (Cologne University), in order to ensure data privacy. OFFIS acts as trust centre and is responsible for development and implementation of the pseudonymisation software, PMV performs plausibility checks and the data preparation following the criteria of the “Good Practice Secondary Data Analysis” [46]. The final data set, not including any identification, cryptograms or any ID-number which can be traced back to the original data sources, will be forwarded to the Institute for Social Medicine and Health System Research (ISMG) at the Otto von Guericke University, responsible for statistical analyses (Fig. 1).

The data flow and the steps taken to ensure anonymity are currently under review for GDPR-compatibility.

### Exploration of actual long-term needs of survivors (module 2)

In order to approach the problem of needs-based care for childhood and adolescence cancer survivors, we will consider Bradshaw’s stratification of needs (Table 3) [47].

**Table 3:** Types of social needs

Type of social need	Defining group and explanation
Normative need	Experts. A “desirable” standard. Need which an expert or a professional defines as a “need” in any given situation
Felt need	Affected person. Here need is equated with want
Expressed need	Affected person. It is an intention, which will be turned into action
Comparative need	Experts. A measure of need, which is obtained by studying the characteristics of a population in comparison to another

Needs underlie behavioural intent, resulting in a specific health behaviour [48]. To examine normative and comparative needs, we will invite healthcare professionals involved into follow-up care to participate in focus groups. Felt and expressed needs will be studied through episodic narrative interviews [49] with cancer survivors and their informal caregivers.

The interviews will be based on two interconnected theoretical approaches [50], the theory of planned behaviour (TPB) [51] and the stereotype priming model (SPM) [52]. SPM can help to overcome the limitations of a purely TPB-based approach as it addresses primers that affect behaviour at a subconscious level and underlie most action intentions [50]. TPB-related predictors (attitude, subjective norm, perceived control) will be assessed with help of an interview [53], and underlying stereotype-primes are going to be investigated from the results of a free associations task. Accordingly, the interview will begin with a free association task, and continue with open-ended and Likert-scale questions. We developed three different interviews guides each adapted for a different age group (12–18, and 18 +). We will also suggest a separate interview guide for cancer survivors’ informal caregivers (up to age of 25). A separate block of questions will focus on the specific needs of transition patients. A specifically developed glossary will be shown to interviewees to improve the comprehension of key concepts.

After a pilot phase, up to 30 patients and their relatives will be recruited either for in-person or video-supported interviews through advertisements in magazines, on target-group specific websites, or through partners at participating clinics. The recruitment process will be stratified according to age, diagnoses, and length of follow-up. Childhood or adolescence cancer survivors and their informal caregivers will be interviewed separately in order to minimise mutual influence on each other’s answers.

Focus group discussions will be organised to explore the level of knowledge about guidelines, as well as attitudes towards and desires for optimal care. Challenges of intersectoral and interdisciplinary cooperation among different groups of healthcare professionals will be also included into the discussions. We expect to organise four focus group discussions with up to eight participants each. The focus groups will be performed in either of two formats: as a video conference or as a face-to-face event. A basis for the focus groups discussions will be an instrumental case study (a standardized follow-up history of a childhood or adolescence cancer survivor) [54].

All interviews and focus groups discussions will be audio-recorded, professionally transcribed and analysed using the Framework method [55].

### Evaluation of selected guidelines (module 3)

We will evaluate selected follow-up guidelines and adherence to them in detail. For this analysis, the observed care of patients after cancer in childhood and adolescence will be compared to corresponding German guidelines and aftercare plans.

We have selected the following types of cancers for this evaluation: neuroblastoma (NB), non-Hodgkin’s lymphoma (NHL), intracranial germ cell tumours (KZT) and lymphatic leukaemia (LL) to consider both solid (NB, KZT) and systemic (NHL, LL) cancer types; malignancies typically diagnosed at an early age (NB, LL) or much later (NHL, KZT); as well as rare (KZT) and frequent (LL) entities. For all four entities, it is possible to obtain intent-to-treat data for most patients in collaboration with the dedicated clinical trials and UK Bonn. Together, these entities account for around 35% of all childhood and adolescent cancers (LL: 22%, NB 6%, NHL 6%, KZT 1%) [1]. We will analyse two types of late effects for these cancer types: audiological late effects, observed to occur in 26% to more than 90% of survivors [55, 56] depending on respective clinical factors, selected groups and the respective endpoint (typically related to platin-based substances), and cardiological late effects (typically related to anthracyclines). Cancer survivors generally reported heart failure, myocardial infarction, pericardial disease, or valvular abnormalities significantly more often than their siblings [56–58].

Results of the whole study will be discussed with stakeholders (policy makers and healthcare providers) in order to suggest practically applicable improvements to selected follow-up guidelines through creation of a clear structure for an assessment of barriers on the basis of the Theoretical Domains Framework [59], thus enabling behavioural change in childhood and adolescence cancer survivors.

## Discussion

The suggested study design of modules 1 and 3 is aiming to provide representative evidence about late effects of childhood cancer based on the largest available set of unselected health care data in Germany. The combined, synergistic use of three data sources – claims data from statutory health insurance companies, GCCR data and clinical treatment data – via a patient-based data linkage is the attempt to overcome limitations of existing research. The linkage of GCCR data and claims data from statutory health insurance companies allows for the first time to gather and analyse information about the current health state of former childhood cancer survivors, even if an individuals’ former cancer diagnoses are not captured in the current health insurance companies’ databases. This is particularly relevant since age-associated health problems in childhood cancer survivors tend to occur earlier and at a higher rate than in the general population (“accelerated aging”) [60]. The availability of a comparison group will allow us to distinguish age-related problems from late effects of paediatric cancer. For a subgroup of selected diagnoses, details about the intended therapy can be included into the analyses. The description of the physical and psychological late effects caused by the disease or its treatment for a selected group of cancer diagnoses will help to identify potential limitations and, consequently, suggest improvements to the existing follow-up guidelines. Moreover, it might show existing gaps between the follow-up guidelines and its implementation, thus giving a room for further improvement of follow-up care to balance “medical and psychosocial health with socio-economic hardship” [61] in paediatric cancer survivors.

These findings are going to be supplemented with methodologically well-founded information from interviews with patients, informal caregivers, and healthcare professionals in the cross-sector care of childhood and adolescence cancer survivors. The case study approach will give an insight into survivorship burden: survivors’ own story added with observation of follow-up appointments done by one of the researchers aims to objectively assess current state of provided follow-up care. Interviews will provide deeper understanding of actual needs of survivors and their perception of provided follow-up care. Further discussion of case studies in healthcare professional focus groups will allow to consider follow-up care from standpoint of different participants and will yield specific suggestions about its organisation. The expected findings from these newly combined sources might lay the groundwork for an improvement of existing long-term follow-up guidelines, possibly lead to new additions to them as well as enable creation of new organisational forms of follow-up care.

### Strengths and limitations

The multi-method approach applied in this study attempts to overcome the limitations of previous research originated mainly from a single data source or conducted for a limited follow-up period after a treatment end.

With a significant number of statutory health insurance companies being involved, we will be able to study a representative population of childhood or adolescence cancer survivors. The unique data linkage will enable us to describe and analyse different types of cancer and compare childhood or adolescence cancer survivors’ health condition to a general population. This type of matching of a registry cohort with health claims data has never been done before in Germany, and it will help us to avoid self-selection and recall biases, which frequently occur in studies relying mainly on self-reported information. The strength of the latter lies in their attention to detail and additional possibility of stating very specific questions. Comparison of our results to such studies can help to quantify the size and direction of such biases and potentially help to correct these to some extent in the future.

In addition to the quantitative analysis of the linked data, our study will also include the perspective of patients, informal caregivers and care providers.

We are aware that our study is not free from limitations. We will be using claims data from the years 2017 to 2022. Thus, we will be able to make statements about current prevalence, but not incidence or time trends. Cohort effects will mix with age effects to a certain extent. We only capture claims for patients below the age of approximately 45, which will limit our ability to make statements about differences in the prevalence and onset of age-associated diseases, as they would generally occur much later in life, at least in the comparison group.

Claims databases contain information limited to diagnoses and treatments for which specific billing codes currently have been assigned. Services paid by the patients are not documented (e.g., over-the-counter drugs). Moreover, diagnoses not requiring diagnostic procedures and not resulting in a billable treatment may be underreported.

Because most medications and procedures have many indications, it can be difficult to clearly interpret the diagnosis that underlies a given prescription / procedure.

With regard to the interviews, respondents might exhibit a social desirability bias affecting their answers. Since participation in the survey will be voluntary, a selective non-response can be expected. As the interviews and focus group discussions will be partially conducted using a video conference system, it might be difficult to establish a trusting relationship with the interviewees due to the limited non-verbal communication. This challenge might be addressed by conducting a phone call beforehand and thus providing the interviewees with detailed information about the study.

Moreover, we are not specifically considering socio-economic side of follow-up care provision, but quantified results in terms utilization of outpatient and inpatient care may be later used for economical quantification of paediatric cancer burden in Germany.

### Perspectives

The results of this mixed-methods study will provide important insights which then could be directly applied to better understand, append, improve and extend existing follow-up guidelines and to develop new models of care for childhood and adolescence cancer survivors in Germany. The main objective is to reduce the number of subsequent hospitalizations and ultimately improve the quality of life for childhood or adolescence cancer survivors through improved adherence to such guidelines by the healthcare system and patients alike. At the end of the study, we plan a workshop with the main stakeholders in the area of cancer follow-up, in order to discuss how our results can be integrated in the existing follow-up routines or how they might be used to suggest new approaches to follow-up care.

## Data Availability

De-identified participant data from this research will be shared upon reasonable request with the corresponding author, if it is not contradicting the German date regulations.

## Abbreviations

GCCR: German Childhood Cancer Registry
PR: Prevalence ratio
ICD-O: International Classification of Diseases for Oncology
ICCC: International Classification of Childhood Cancer
TPB: Theory of planned behaviour
SPM: Stereotype priming model
NB: Neuroblastoma
NHL: Non-Hodgkin’s lymphoma
KZT: Intracranial germ cell tumours
LL: Lymphatic leukaemia
